# The Monitoring and Management of an Operating Environment to Enhance the Safety of a Container-Type Energy Storage System

**DOI:** 10.3390/s23104715

**Published:** 2023-05-12

**Authors:** Hye-Yeon Park, Jin-Wook Lee, Sung-Won Park, Sung-Yong Son

**Affiliations:** 1 Department of Next Generation Smart Energy System Convergence, Gachon University, Seongnam-si 13120, Republic of Korea; 2 Department of Electrical and Electronic Engineering, Youngsan University, Yangsan-si 50510, Republic of Korea

**Keywords:** ESS, algorithm, electrochemical model, monitoring, safety, temperature, humidity

## Abstract

The implementation of an energy storage system (ESS) as a container-type package is common due to its ease of installation, management, and safety. The control of the operating environment of an ESS mainly considers the temperature rise due to the heat generated through the battery operation. However, the relative humidity of the container often increases by over 75% in many cases because of the operation of the air conditioner which pursues temperature-first control. Humidity is a major factor which can cause safety issues such as fires owing to insulation breakdown caused by condensation. However, the importance of humidity control in ESS is underestimated compared to temperature control. In this study, temperature and humidity monitoring and management issues were addressed for a container-type ESS by building sensor-based monitoring and control systems. Furthermore, a rule-based air conditioner control algorithm was proposed for temperature and humidity management. A case study was conducted to compare the conventional and proposed control algorithms and verify the feasibility of the proposed algorithm. The results showed that the proposed algorithm reduced the average humidity by 11.4% compared to the value achieved with the existing temperature control method while also maintaining the temperature.

## 1. Introduction

An energy storage system (ESS) is a system that has the flexibility to store power and use it when required. An ESS can be one of the solutions to mitigate the intermittency effect of variable renewable energy (VRE), such as photovoltaic and wind power [1,2,3]. An ESS is often implemented as a container-type package with an air conditioning system owing to the ease of installation and maintenance. However, the ESS generates heat through the oxidation reduction in ions in the battery during charging and discharging, increasing the battery temperature [4]. An increase in battery temperature reduces performance and shortens battery life [5]; therefore, battery health monitoring and management are important. Determining the state of a battery is essential for estimating its safety and lifespan. In [6], a methodology for battery life prediction and management based on artificial intelligence (AI) is discussed. Ali et al. [7] analyzed various methods for estimating battery health by classifying them into two types: experimental and model-based estimation. In addition, various methods, such as advanced sensing, big data, and multi-agent decision-making techniques are predicted to be used in the future. In [8], various aging and thermal models based on electrochemical models were investigated, and the required parameterization methods were summarized. In [9], the effects of the aging behavior of lithium-ion batteries was measured using an electrochemical-based model. The indoor temperature of the ESS container can be controlled to maintain the battery temperature below the target temperature. Generally, economical and simple forced air convection systems (FACS) are used to manage the indoor temperature of ESS containers [10]. FACS is a method of forcibly moving air for heat transfer of the fluid and installing an air conditioner in the ESS container to manage the battery temperature by controlling the room temperature [11].

Apart from indoor temperature, indoor humidity is highly correlated with battery safety problems. The Pacific Northwest National Laboratory (PNNL) and International Business Machines Corporation (IBM) consider humidity as an ESS operating environment factor and recommend an appropriate range [12,13]. In addition, IEC 62933-4-3, an international standard for ESS system protection, suggests management standards for factors of the ESS operating environment, such as temperature, humidity, and dust [14]. However, in an actual ESS operating environment, the focus is on indoor temperature management, and consideration of humidity is relatively insufficient, especially for a container-type ESS.

The humidity in an ESS container can increase owing to the operating characteristics of the air conditioner. When the indoor temperature reaches the set value of the air conditioner, the frequency of the inverter is adjusted to reduce the number of rotations of the outdoor unit motor, thereby reducing the indoor temperature changes and power consumption. Therefore, the output delivered to the compressor to condense the refrigerant is reduced, and the air temperature from the indoor unit increases. In this state, the amount of moisture condensed on the surface of the air conditioner evaporator increases. At this time, the fan in the indoor unit continues to operate, causing the moisture on the evaporator to evaporate into the ESS container and increase the indoor humidity [15]. When the indoor humidity increases, ventilation can be used when the outdoor humidity is low. However, ESS is often designed as a sealed container to prevent the spread of a fire; therefore, humidity management through ventilation is limited [16]. However, even if the ESS container is sealed, it is very difficult to block air inflow from the outside completely. Xiong et al. [17] analyzed office air permeability using CO_2_ concentration and humidity ratio data, while Tanyer et al. [18] investigated the airtightness performance and consequent energy efficiencies of four types of container houses (CHs). Although the airtightness of the CH junction was improved, it was confirmed that the condensation problem still occurred. In addition, people should be able to access the ESS container easily for maintenance. Therefore, humidity needs to be managed through control.

If the indoor humidity of an ESS container is maintained at a high level, moisture, dew condensation, and drying are repeated, and the surrounding dust can stick to the battery [19]. This may cause the insulation performance to deteriorate and cause short-circuit accidents [20,21,22]. Jang et al. [23] designed test evaluation items considering field conditions such as condensation and identified dust at the ESS fire site survey. Based on the test results, condensation and drying occurred repeatedly in the battery module. As a result, it was confirmed that a fire could occur because the dust was pressed together and the insulation was broken in the ground part between the battery cell and the module enclosure. These problems can lead to battery fire. Therefore, both indoor temperature and humidity should be properly managed. Indoor humidity management issues are addressed in various areas, such as server rooms, data centers, and buildings. A microcontroller-based fuzzy logic was designed in [24] to control the indoor temperature and humidity of the server room and was used to control the set temperature and operation mode of the air conditioner. Novel dew point evaporative cooling was proposed in [25] with air-carrying energy radiant air conditioning and a vacuum membrane-based dehumidification (DAV) cooling system. This system does not use any refrigerants, is eco-friendly, and it can be applied to high-humidity environments. The DAV can operate in high-temperature conditions of supply air, thereby saving energy by increasing the supply of air temperature and relative humidity. A model predictive control (MPV) method was proposed in [26] to control the temperature and humidity coupled with an air conditioner system. A non-linear control design method that simultaneously controls the temperature and humidity of a barn building during the summer and winter for domestic animals sensitive to climatic conditions was proposed in [27]. The improvement and performance of temperature and humidity independent control (THIC) in terms of building application was reviewed in [28]. Hardware-based combined (HWBD) and software-based combined (SWBD) control methods, which use additional equipment or systems that consider the lack of humidity management equipment in small and medium buildings compared to large buildings, were introduced in [29].

Recently, ESS monitoring and management have evolved in various forms, including the accurate measurement or prediction of the temperature inside a battery, monitoring through a combination of IoT sensors and digital twin models, and cloud-based monitoring and management technology. Regarding high-precision measurements, to monitor the conductivity of the battery coolant, an integrated interdigital platinum electrode and thermal-resistant micro-sensors were proposed and implemented as a microelectromechanical system (MEMS) [30]. Wei et al. [31] proposed a new smart battery configuration called a thermal model-based low order bonding observer by inserting a distributed fiber optical sensor (DFOS) into a lithium-ion battery to monitor the state accurately. In [32], a digital twin application technology for energy storage was reviewed, and the digital twin functionality, architecture, and research directions for future commercialization were proposed. Qin et al. [33] proposed digital twin solutions that could estimate battery SOH using real-time data and update physical battery models. Tran et al. [34] reviewed the concepts and designs of cloud-based smart BMS to illustrate their features and benefits for future battery applications. In [35], a cloud battery management system was proposed, and a digital twin for battery systems was developed. In [36], a cyber hierarchy and interactional network (CHAIN) framework that leverages end–edge–cloud architecture for a cloud-based BMS was proposed.

Existing studies have focused on temperature and humidity management methodologies and control algorithms for air-conditioning facilities in various environments, such as rooms or livestock sheds in buildings. However, the operating environments and installation conditions in these studies were different from those of ESS containers. In buildings, additional dehumidification facilities and auxiliary devices are used to manage the indoor temperature and humidity simultaneously; however, they are not cost-effective for ESS containers. In addition, these approaches have limitations in that additional costs are required or new technology should be reflected in the early design stage. This study proposes a cost-effective method for managing ESS based on existing systems. For this purpose, temperature and humidity sensors, air conditioner motion sensors, and control devices were installed inside an ESS container, and data collection and control systems were constructed. The humidity problem was analyzed according to the operating characteristics of an air conditioner, and a rule-based air conditioner control algorithm was proposed. The algorithm controls the air conditioner by considering the target temperature and humidity management of the ESS container.

The remainder of this paper is organized as follows. Section 2 deals with issues related to ESS system operation, the humidity problem during operation, and control system and interface construction. Section 3 deals with the modeling and control algorithm used to solve the humidity problem. Section 4 presents the case study, and Section 5 concludes the paper.

## 2. Materials and Methods

### 2.1. Container-Type ESS and Operating Environment

#### 2.1.1. Container-Type ESS

**Figure 1:**
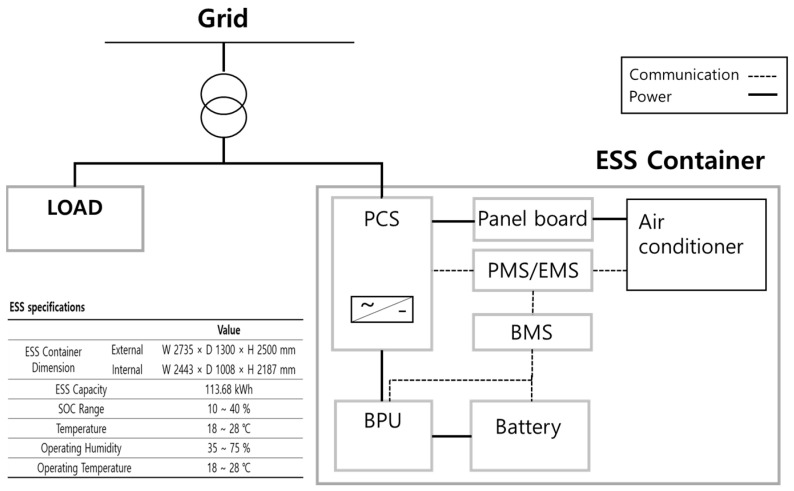
Concept diagram of a generic ESS container.

The general configuration of an ESS container is shown in Figure 1. It consists of a power conversion system (PCS), battery protection unit (BPU), battery management system (BMS), and battery. The PCS converts AC power to DC power during charging and vice versa during discharging. The DC power converted from the PCS flows through the BPU and is stored in the battery. The BPU is a battery protection device which prevents damage and failure of a battery during over-charging, over-discharging, over-current, and battery over-temperature situations. The BPU implements protection control based on a built-in algorithm when the BMS detects a problem in the battery. The BMS monitors the voltage and current status in the cells in the battery module and blocks the external switch through the BPU when problems, such as over-current or short circuit, occur.

#### 2.1.2. The Humidity Problem in a Container-Type ESS

**Figure 2:**
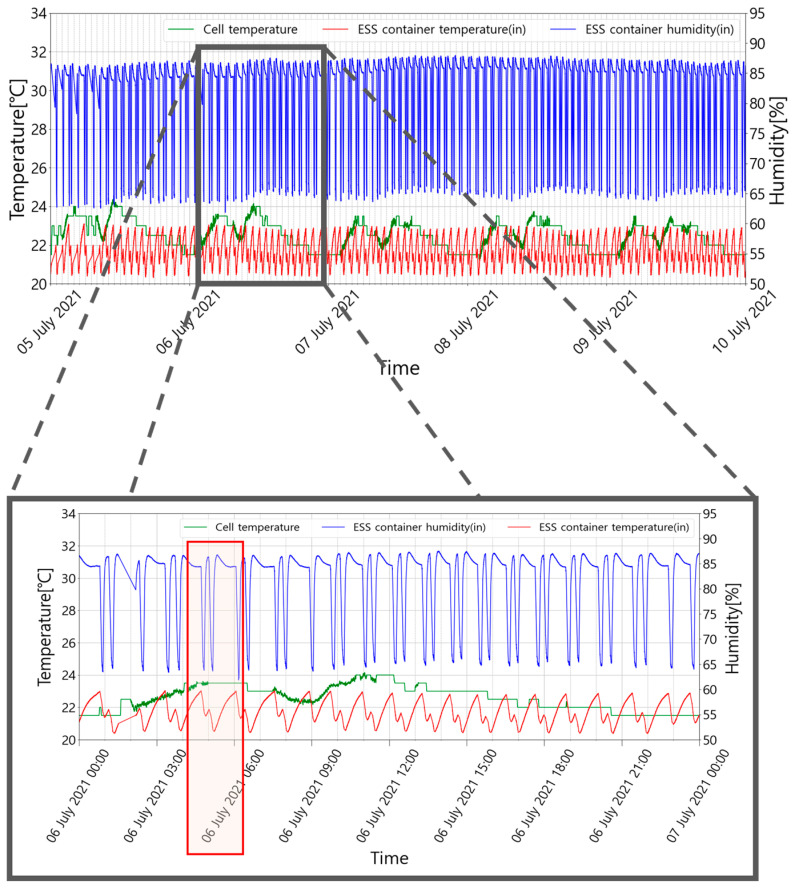
Changes in humidity and temperature during the operation of the air conditioner in the ESS container.

In general, an ESS has quality assurance standards for the operating environment, and each manufacturer provides an appropriate temperature and humidity management range [37]. The operating environment of an ESS must be managed within the operating range provided by the manufacturer. It is recommended that the ESS container used in this study be operated at 35~75% humidity and 18~28 °C. Figure 2 shows an example of the relative humidity, temperature of the container, and battery cell temperature during summer. In this example, the set temperature of the air conditioner inside the ESS container was set to 21 °C. As shown in Figure 2, the indoor temperature of the ESS container was maintained at 20.3~23.1 °C for five days (5 July 2021 00:00 to 9 July 2021 24:00); however, the maximum humidity was over 85%, exceeding the appropriate operating range.

The general method for temperature management inside an ESS container is to maintain the room temperature near the set temperature by operating the air conditioner at all times. However, this method can cause problems if there is an unexpectedly high indoor humidity. Recently, inverter-type air conditioners are being widely used in ESS containers owing to their high energy efficiency. The air conditioner enters the blowing mode when the indoor temperature reaches the set temperature. Then, the compressor power and fan speed of the outdoor unit of the air conditioner decrease, while the indoor unit fan continues to operate. Subsequently, the moisture condensed on the surface of the evaporator coil evaporates into the ESS container, causing a rapid increase in humidity [38]. This problem can be seen in the magnified grey box in Figure 2. The grey box in Figure 2 shows the operating results of ESS for one day (7 July 2021 00:00 to 7 July 2021 24:00). The initial temperature is 23 °C, and the air conditioner is activated in the cooling mode to meet the target set temperature of 21 °C. The indoor temperature decreases to the target temperature within approximately 30 min. Then, the relative humidity rapidly decreases to 65% owing to the dehumidification effect of overcooling [39]. However, the moisture condensed on the surface of the evaporator coil flows into the container owing to the air conditioner fan, and the internal relative humidity sharply increases to over 85% within a few minutes. This can be found in the red box in Figure 2. Since the air conditioner is controlled by considering only the indoor temperature, it is not managed properly, even if the humidity exceeds the standard range.

### 2.2. Monitoring and Control System Design

#### 2.2.1. System Configuration

**Figure 3:**
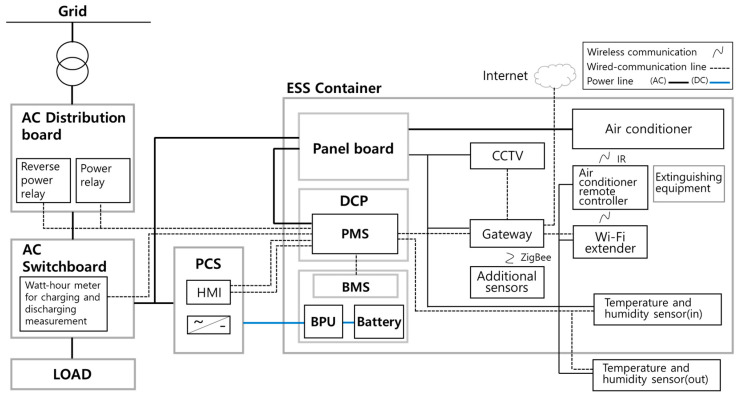
Schematic of the ESS container.

Figure 3 shows the schematic of the ESS container used in this study. The ESS is directly connected to the plant. The ESS container has internal dimensions of 2443 mm width, 1008 mm depth, and 2187 mm height and is used for the peak shaving of plant loads. It is composed of a cylindrical lithium-ion battery with a capacity of 113.68 kWh, BPU, PCS, a DC distribution board, an air conditioner, fire extinguishing equipment, CCTV, sensors, and a communication system. It is designed with a closed container structure that facilitates thermal management of the battery and effectively prevents the spread of fire.

The battery rack in the container consists of 11 battery modules and 1 BPU. Each battery module is equipped with a module BMS. The measurement data collected by the BMS module are sent to the BMS, which controls the charging and discharging of the ESS. An air conditioner is installed inside the ESS container for cooling purposes. Table 1 lists the specifications of the ESS and operating environment.

**Figure 4:**
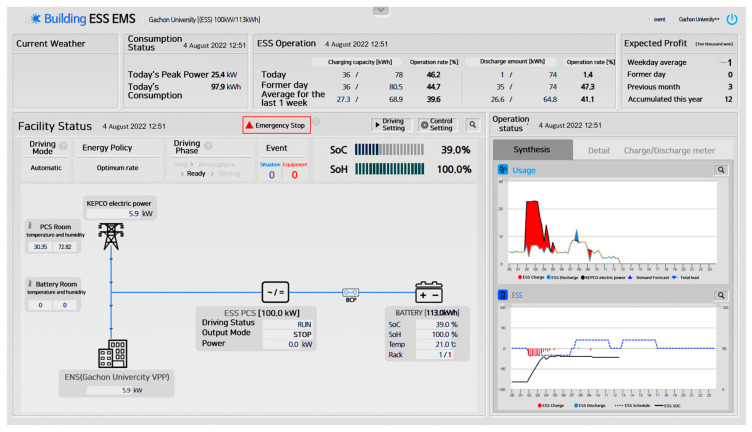
ESS container monitoring system.

Figure 4 shows a screenshot of the ESS monitoring system. The monitoring parameters include the charge–discharge schedule, battery voltage and current, state of charge (SOC), state of health (SOH), battery cell temperature, AC 3-phase voltage and current of the PCS input side, and the DC voltage and current of the PCS output side.

#### 2.2.2. Monitoring and Control Systems

**Figure 5:**
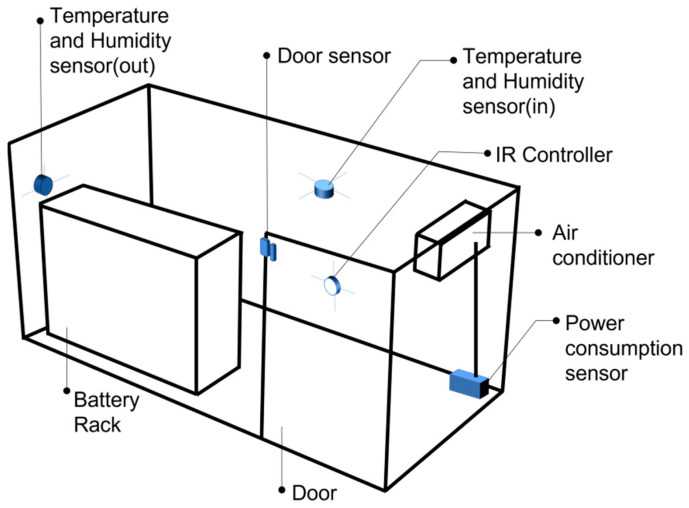
Sensor locations inside the ESS container.

Figure 5 shows the locations of the sensors installed inside and outside the ESS container. The ESS includes PCS-side sensors that measure power data, such as voltage, current, and power. In addition, it includes BMS-side sensors that measure the internal data of the battery, such as battery cell temperature, battery internal voltage, current, and power. In this study, internal and external temperature sensors, humidity sensors, air conditioner power consumption sensors, and door open sensors were also installed. Furthermore, an infrared (IR) controller was installed to manage the air conditioner.

**Figure 6:**
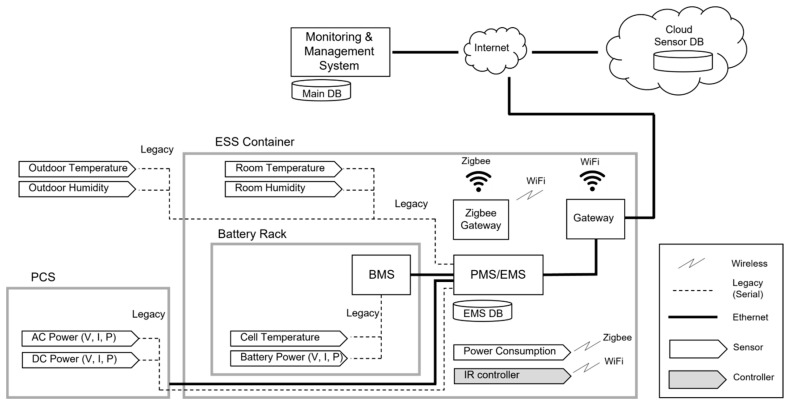
Schematic of the monitoring and control system.

Figure 6 shows the schematic of the monitoring and control systems. The sensor data from the PCS and rack battery are stored in the energy management system (EMS) database (DB) inside the power management system (PMS)/EMS through serial communication. The indoor–outdoor temperature and humidity sensor data are stored in the EMS DB through serial communication. The sensor data to control the ESS container’s indoor temperature and humidity are stored in the cloud sensor DB through Zigbee communication. Power consumption and status data that measure the condition of the air conditioner and door open–close status data that determine whether ventilation is present are stored in the cloud sensor DB through the Zigbee gateway. The cloud DB is configured to work with the main DB again. The EMS DB is linked to the main DB using network communication through a WiFi gateway. The PMS/EMS operates every two seconds, and the indoor temperature of the ESS container and humidity data are collected every four seconds. The operation algorithm is executed every four seconds. The power consumption and status of the additionally installed air conditioner are set to be collected when the value changed. The IR controller can control the operation of the air conditioner in the ESS container using WiFi communication.

## 3. Modeling and Algorithms

### 3.1. Temperature Model

**Figure 7:**
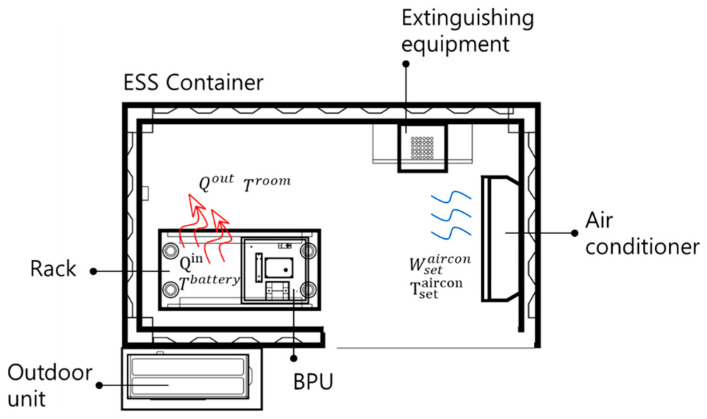
Air and heat flow through ESS operation.

Figure 7 shows the flow of the heat and air according to the ESS operation. $Q_{in}$ is the amount of heat generated by the ESS operation; $Q_{out}$ is the amount of heat transferred from the battery to the ESS container through the ESS operation; The red arrow shows the direction of heat dissipation from the battery to the indoor; $T^{battery}$ is the battery cell temperature [°C]; $T^{room}$ is the ESS container indoor temperature [°C]; $T_{set}^{aircon}$ represents the air conditioner temperature setting; $W_{set}^{aircon}$ is the air conditioner wind speed mode setting; and the blue arrow shows the direction of the wind.

In this study, the lump-sum capacitance model was used to obtain the battery cell temperature prediction value used in the rule-based air conditioner control algorithm. Here, the necessary parameters, time constant *τ* and heat capacity *C*, are calculated using Equations (1)–(5), and their values are applied to Equations (6) and (7).

Equation (1) represents the amount of heat $Q_{in}$ generated through the ESS operation. Here, *η* is the ESS operating efficiency [%], and *P* is the ESS output [W]. Equation (2) represents the heat $Q_{out}$ transferred from the battery to the ESS container through the ESS operation. Here, $R^{th}$ represents the thermal resistance [°C/W].

(1) $$Q_{in}=\left(1-\eta \right)\times P,$$

(2) $$Q_{out}=\frac{\left(T^{battery}-T^{room}\right)}{R^{th}},$$

When the lumped model is applied to the ESS, the temperature change in the ESS can be expressed using Equation (3).

(3) $$Q_{in}-Q_{out}=C\frac{T^{battery}}{dt},$$

By substituting Equations (1) and (2) into Equation (3), Equation (4) can be obtained as:

(4) $$C\frac{T^{battery}}{dt}+\frac{\left(T^{battery}-T^{room}\right)}{R^{th}}=\left(1-\eta \right)\times P,$$

In Equation (4), $T^{room}$ is constant as the container temperature is set to a specific value, and $\frac{T^{battery}}{dt}$ can be written as $\frac{T^{battery}-T^{room}}{dt}$. To simplify the formula $\;T^{battery}-T^{room}$, $T^{diff}$ =$\;T^{battery}-T^{room}$ has been defined. And the relationship between thermal resistance, time constant, and heat capacity, which is defined as $R^{th}=\frac{\tau }{C}$ [40], is used. With these newly defined variables, Equation (4) can be expressed as Equation (5).

(5) $$C\frac{dT^{diff}}{dt}+C\times \frac{T^{diff}}{\tau }=\left(1-\eta \right)\times P,$$

Equation (5) can be expressed as Equation (6) when *P* ≠ 0, which implies that the ESS is operating. When *P* = 0, the ESS does not operate, and Equation (5) can be expressed as Equation (7), where $T^{0}$ is the initial temperature of the battery.

(6) $$T^{diff}=\frac{\left(1-\eta \right)\times P}{C}\times \tau \times \left(1-e^{-\frac{t}{\tau }}\right),\;\;\;\left(P\neq 0\right)\;$$

(7) $$T^{diff}=T^{0}e^{-\frac{t}{\tau }}\;,\;\;\;\left(P=0\right)$$

The predicted battery temperature $T_{forecast-on}^{battery}$ and $T_{forecast-off}^{battery}$ can be obtained through Equations (6) and (7) for the two situations when the air conditioner of the ESS container is turned on and can be expressed as Equations (8) and (9):

(8) $$T^{battery}=T^{room}+\frac{\left(1-\eta \right)\times P}{C}\times \tau \times \left(1-e^{-\frac{t}{\tau }}\right),\;\;\;\left(P\neq 0\right)\;$$

(9) $$T^{battery}=T^{room}+T^{0}e^{-\frac{t}{\tau }}\;,\;\;\;\;\;\;\;\left(P=0\right)$$

### 3.2. Humidity Model

The absolute humidity $AH^{room}$ is calculated using the indoor temperature $T_{t}^{room}$ and relative humidity of the ESS container $RH^{room}$ as shown in Equation (10). Here $SMC_{T}$ is the amount of saturated water vapor when the indoor temperature of the ESS container is *T*.

(10) $$AH^{room}=\frac{RH^{room}\times SMC_{T}}{100},$$

The target absolute humidity $AH_{set}^{deh}$ can also be calculated using the target indoor temperature $T_{set}^{room}$ and the target relative humidity of the ESS container using Equation (10).

### 3.3. Control Strategies

**Figure 8:**
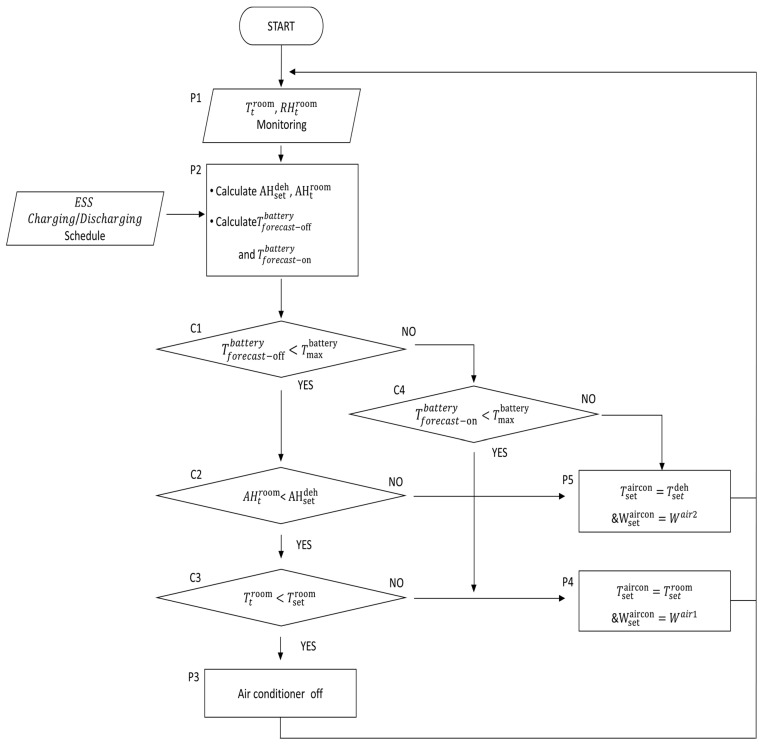
Rule-based air conditioner control algorithm.

As shown in Figure 8, the rule-based air conditioner control algorithm used thermal and humidity models to determine the operation by determining the compliance with constraints based on real-time collection data and operational information. In this algorithm, the absolute humidity was calculated using the ESS charge–discharge schedule, indoor temperature, and humidity data. The absolute humidity to be achieved is computed using the target temperature and humidity. The humidity of the ESS container should remain within the target absolute humidity range while maintaining the battery temperature within a safe operating range.

The battery temperature increases as per the operation of the ESS, which affects the battery life. The battery temperature can be predicted using the ESS charging–discharging schedule and environmental temperature.

The steps of the rule-based air conditioner control algorithm are as follows:

1. [P1] Measure $RH_{t}^{room}$ and $T_{t}^{room}$ of the ESS container using a sensor.
2. [P2] Calculate the $AH_{t}^{room}$ using the measured $RH_{t}^{room}$, and $T_{t}^{room}$. And calculate the $AH_{set}^{deh}$ using $RH_{set}^{deh}$, and $T_{set}^{room}$ with the humidity model. Additionally, calculate the steady-state battery temperature using the temperature prediction model to manage the battery temperature. Here, $T_{forecast-on}^{battery}$ and $T_{forecast-off}^{battery}$ are the steady-state temperatures of the battery when the air conditioner is on and off, respectively. At this time, the charge–discharge operation schedule is reflected to consider the heating effect.
3. [C1] Check whether the safety range is maintained by comparing the battery temperature prediction value with the battery temperature upper limit value, $T_{max}^{battery}$, through on–off operation of the air conditioner. $T_{forecast-off}^{battery}<$ $T_{max}^{battery}$ indicates that the battery temperature satisfies the management range, even when the air conditioner is off.
4. [C2] Compare $AH_{t}^{room}$ and $AH_{set}^{deh}$ to manage the indoor humidity of the ESS container based on absolute humidity. $AH_{t}^{room}<AH_{set}^{deh}$ indicates that additional humidity control is not required.
5. [C3] If $T_{t}^{room}<T_{set}^{room}$, air conditioning is not required.
6. [P3] Turn off the air conditioner when additional air conditioning is not required.
7. [C4] When the air conditioner is not being operated, check whether the predicted temperature exceeds the temperature range compared to the upper limit of the temperature maintenance range, $T_{max}^{battery}$. $T_{forecast-on}^{battery}$< $T_{max}^{battery}$ indicates that the battery temperature satisfies the management range when the air conditioner is turned on.
8. [P4] Set the air conditioner set temperature $T_{set}^{aircon}$ to the target indoor temperature $T_{set}^{room}$ because air conditioner operation is required and there is no need for separate humidity management. In addition, set $W_{set}^{aircon}$ (set wind speed of the air conditioner) to $W^{air1}\left(\mathrm{lowest}\;\mathrm{wind}\;\mathrm{speed}\right)$. Simultaneously, set the wind speed mode to $W^{air1}$ because it minimizes the re-inflow of moisture from the evaporator into the ESS container.
9. [P5] Set $T_{set}^{aircon}$ to the lowest set temperature $T_{set}^{deh}$ because active dehumidification or additional battery temperature management is required. Set the wind speed mode $W_{set}^{aircon}$ to a high wind speed, $W^{air2}$, to operate the air conditioner.

## 4. Results

### 4.1. Experimental Verification

#### Case Study Setting

To verify the feasibility of the proposed air conditioner-based indoor temperature and humidity control algorithm, the testing was performed in an ESS container environment. For the ESS used in this study, an operating environment within a temperature range of 18~28 °C and relative humidity of 35~75% is recommended. The case study consisted of two cases depending on the application of the proposed air conditioner control algorithm. Case 1 consisted of an ESS operating environment that considered only conventional temperature control. The experiment was conducted by setting the air conditioner set temperature $T_{set}^{room}$ inside the container to 21 °C and setting the mode setting variable $W_{set}^{aircon}$ to automatic. In Case 2, the proposed algorithm was applied to configure an air conditioner ESS operating environment considering both temperature and humidity. Additionally, the proposed algorithm was applied by considering the parameters $T_{set}^{room}$, $RH_{set}^{deh}$, $T_{max}^{battery}$, $T_{set}^{deh}$, and $W^{air1}$. $T_{set}^{room}$ and $RH_{set}^{deh}$ were set to 21 °C and 60%, respectively, considering the battery manufacturer’s specifications and seasonal characteristics. $T_{max}^{battery}$ was set to 40 °C, which applies the upper limit of the recommended battery temperature range. $T_{set}^{deh}$ was set to 18 °C, which is the lowest settable temperature of the air conditioner, to induce supercooling of the ESS container indoors to generate a dehumidifying effect. $W^{air1}$ was set to Mode 1, which has the lowest wind speed of the air conditioner. $W^{air2}$ was set to Mode 2, which has the highest wind speed of the air conditioner. *η* was 94%, which was set as the average operating efficiency of the ESS calculated for one year. The input parameter setting values for each case are listed in Table 2.

### 4.2. Case Study Results and Discussion

**Figure 9:**
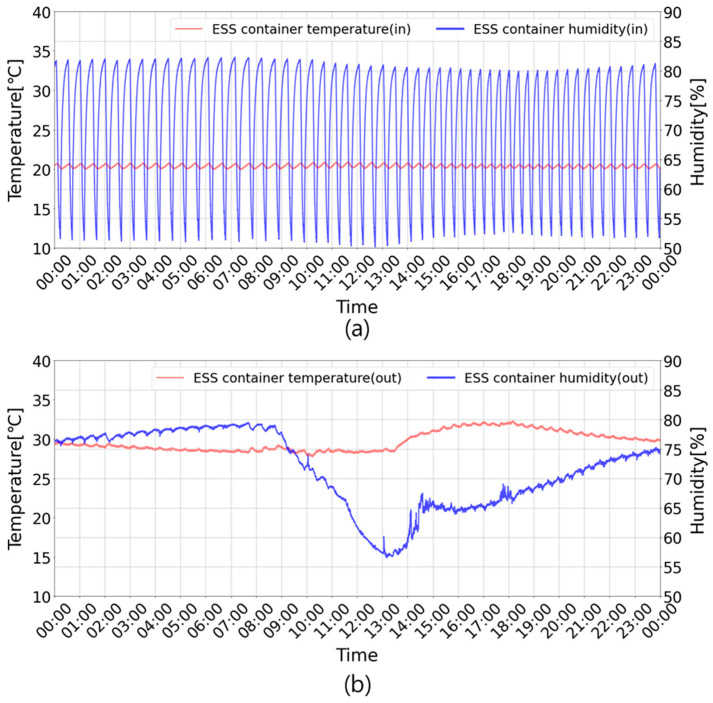
ESS operation results without the proposed control algorithm. Case 1: (**a**) the ESS container’s indoor temperature and humidity; (**b**) the ESS container’s outdoor temperature and humidity.

Each case was operated for one similar consecutive day to verify the effectiveness of the proposed algorithm. Figure 9 shows the results for Case 1. The red and blue lines represent the indoor–outdoor temperature and relative humidity of the ESS container, respectively. During the ESS operation period, the indoor temperature was maintained within 20–20.9 °C, and the indoor humidity was maintained at 50.2–82.3%, while the outdoor temperature was in the range of 27.7–32.3 °C, and outdoor humidity was in the range of 56.6–79.5%. High indoor humidity may corrode the battery and reduce its lifecycle.

**Figure 10:**
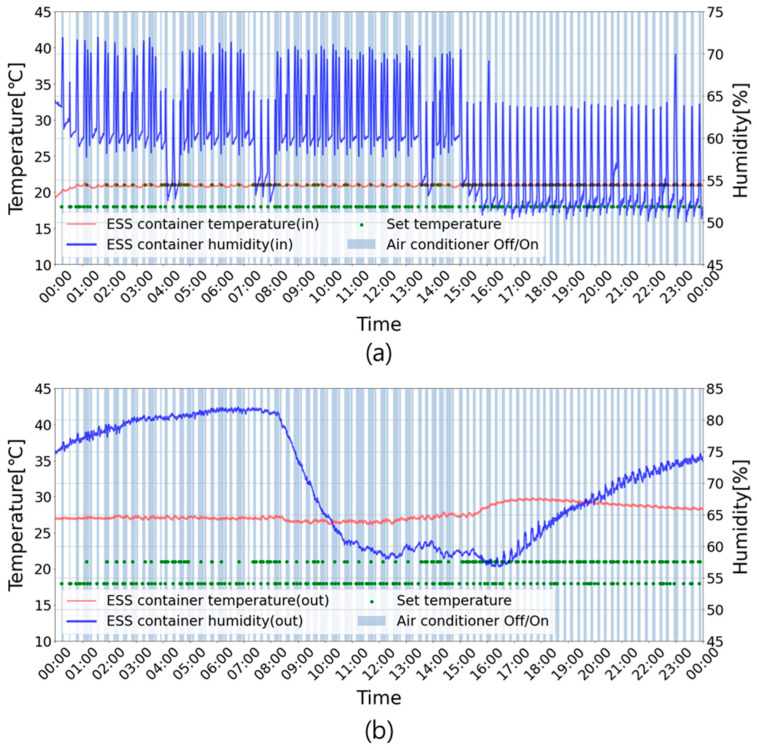
ESS operation results with the proposed control algorithm. Case 2: (**a**) the ESS container’s indoor temperature and humidity; (**b**) the ESS container’s outdoor temperature and humidity.

Figure 10 shows the indoor temperature and humidity measured after setting up the ESS container for Case 2. Light blue bars indicate the on–off status of the air conditioner. During the ESS container operation period, the indoor temperature was maintained in the range of 19.3–21.3 °C throughout; however, the indoor humidity was in the range of 50.1–72%. The outdoor temperature and humidity were in the ranges of 26.1–29.9 °C and 56.7–82.8%, respectively.

While the indoor temperature remained constant during the period of operation, the indoor humidity was divided into approximate ranges of 60–70% and 52–63% during the operation period. This could be caused by the air conditioner operation algorithm related to the indoor and outdoor temperatures and humidity.

**Figure 11:**
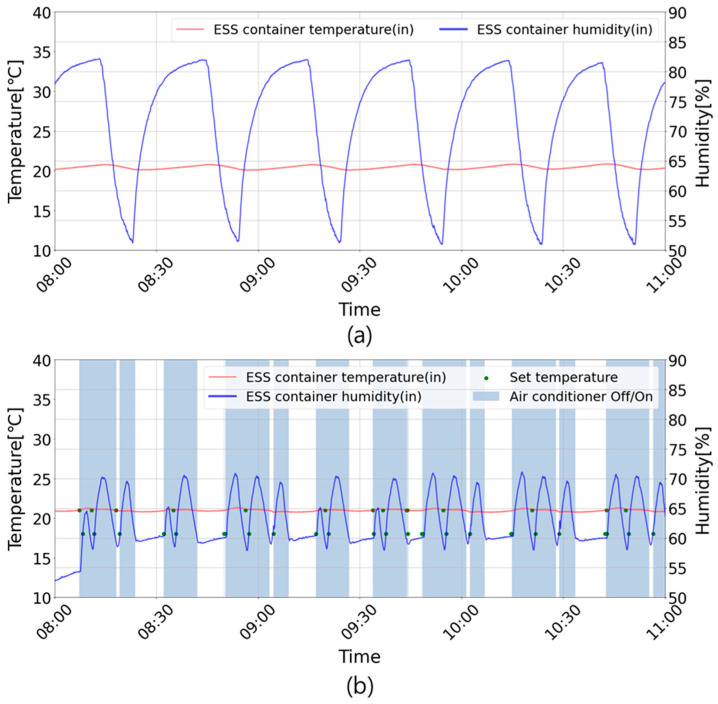
The ESS container’s indoor temperature and humidity for each case during the discharge period: (**a**) the ESS container’s indoor temperature and humidity during the discharge period (Case 1); (**b**) the ESS container’s indoor temperature and humidity during the discharge period (Case 2).

Figure 11 shows the changes in the air conditioner operation, indoor temperature, and humidity during the period of discharge operation in Cases 1 and 2. The red line represents the indoor temperature, and the blue line represents the indoor humidity of the ESS container. The green dots indicate the set temperature of the air conditioner, and the blue bars indicate the on–off status of the air conditioner. Figure 11a shows that the indoor humidity exceeded 80% as the air conditioner continued to operate. Figure 11b shows that the setting temperature of the air conditioner for indoor humidity management changed according to the algorithm, and the operation of the air conditioner was controlled to prevent moisture increase.

**Figure 12:**
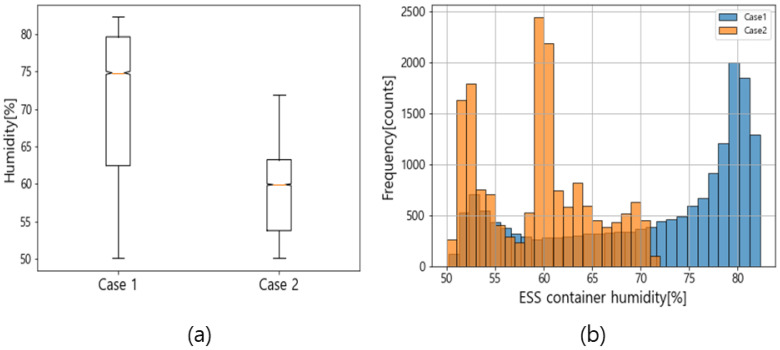
Case study results for the humidity: (**a**) a boxplot of the humidity; (**b**) a histogram of the humidity.

Figure 12a shows the humidity distributions using a box plot for Cases 1 and 2. The upper whisker of the box plot represents the maximum score of the data, and the lower whisker represents the minimum. The upper quartile is the top 75% of values, and the lower quartile comprises the bottom 25%. A comparison between the box lengths of Cases 1 and 2 shows that Case 1 is longer, meaning that the data were more spread out. Figure 12a illustrates that the average daily humidity values in Cases 1 and 2 were 70.8% and 59.4%, respectively, showing a 11.4% reduction in the average humidity using the proposed method. The standard deviations in Cases 1 and 2 were 10 and 5.6, respectively, confirming that the humidity variability also reduced. Figure 12b shows the humidity in Cases 1 and 2 as histograms. A wide distribution, in the 50–80% interval, can be seen in Case 1 in Figure 12b. The frequency range exceeding 1500 was concentrated in the upper 79–81% range. Additionally, distribution in the 50–70% interval can be seen in Case 2 in Figure 12b, and the frequency ranges exceeding 1500 were 51–53% and 59–61%. The proposed algorithm moved the overall distribution range of the humidity to a lower range and lowered the degree of dispersion.

**Figure 13:**
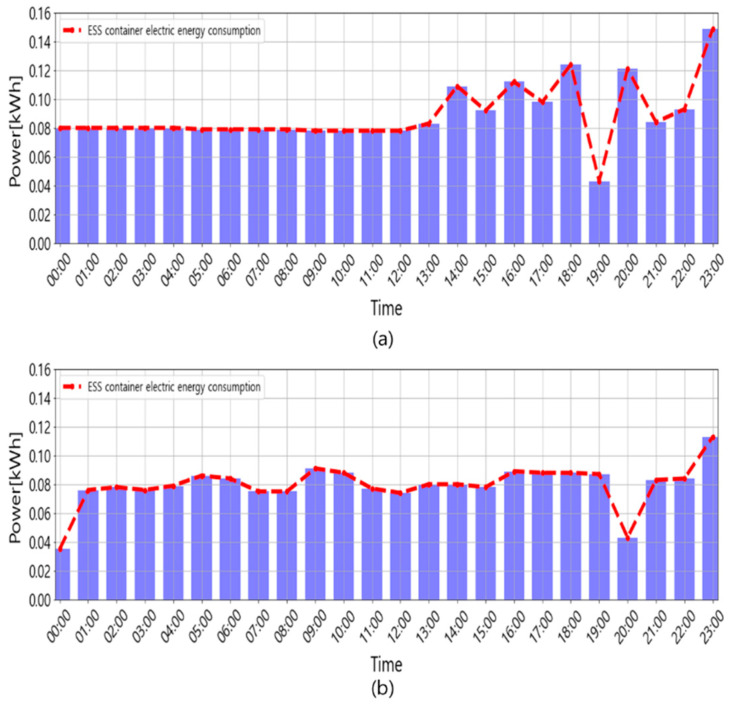
Power usage used to maintain the indoor environment of the ESS container: (**a**) ESS container power usage without the proposed control algorithm (Case 1); (**b**) ESS container power usage with the proposed control algorithm (Case 2).

The cost of maintaining the environment inside the container was mostly used to run the air conditioner. Figure 13 shows the power usage for maintaining the indoor environment of the ESS container. Figure 13a shows that, without the proposed control algorithm, 2.14 kWh of electricity was used for one day, and Figure 13b shows that 1.91 kWh of electricity was consumed for a similar day with the proposed control algorithm, which was 10.7% less than the previous operation. In addition, the product used to implement the control algorithm was SmartRemote, which costs less than $30. However, if a commercially available dehumidifier was used, alongside the initial cost of several hundred dollars, additional electricity would be consumed for its operation. Therefore, the proposed method is more economical compared to the other methods.

## 5. Conclusions

In this study, a sensor-based control system was developed to manage the indoor temperature and humidity of a container-type ESS. The data from the sensors were stored in a database system built to collect and monitor the environmental data of the ESS container. A rule-based air conditioner control algorithm was proposed based on the ESS operation data to solve the problem of the humidity exceeding an appropriate range. The proposed algorithm enhances the operating environment of an ESS container by controlling the operation of commercial air conditioners using thermal and humidity models. Based on the proposed algorithm, the indoor temperature of the ESS container was maintained at 21 °C on average, whereas the relative humidity was suppressed to a value of less than 75%. The average indoor humidity was maintained at 59.4%, which was 11.4% lower than the value achieved with the existing temperature control method. In addition, the standard deviation of the humidity was reduced from 10 to 5.6%. These results indicate that the battery management environment improved, which would consequently slow battery life reduction. In addition, the proposed method can reduce energy consumption by 10.7% compared to the current daily energy consumption without any additional equipment in the case study, resulting in economical operation.

The proposed algorithm controls the set temperature of the air conditioner to manage the indoor temperature and humidity of the ESS; therefore, the ESS container operation environment is improved. However, this requires frequent air-conditioner operation to prevent the reverse flow of moisture from the indoor evaporator. If the air conditioner fan control can be combined with the indoor temperature control of the ESS, better results can be obtained. It is assumed that the air conditioner has sufficient capacity to maintain room temperature throughout the year. If the energy management system considers energy consumption for managing the room environment, the battery operation schedule can be changed. In addition, although this study estimated the battery temperature based on the lump-sum capacitance model, advanced battery models can be used to accurately estimate battery temperature.

This study sought appropriate technology to economically control temperature and humidity by making the most of the existing facilities. Future research should take into account the combination of sealing, control, and energy consumption in indoor environment management and explore how the optimal operation schedule of batteries can be achieved.

## Figures and Tables

**Table 1:** ESS specifications.

		Value
ESS Capacity		113.68 kWh
Rated Capacity		142.42 Ah/4.10 V_dc_
Charge Maximum		40 kW
Discharge Maximum		50 kW
Operating Current	A_dc_ min	48.7 A_dc_
	A_dc_ max	67.3 A_dc_
Voltage Range	V_dc_ min	623.7 V_dc_
	V_dc_ max	811.8 V_dc_
Operating Temperature		23+/5 °C
Air Conditioner Cooling Capacity		2.8 kW
ESS Container Dimension	External	W 2735 × D 1300 × H 2500 mm
	Internal	W 2443 × D 1008 × H 2187 mm

**Table 2:** Input parameters in the case studies.

Parameter	Case 1	Case 2
$T_{set}^{room}$ [°C]	21 [°C]	21 [°C]
$RH_{set}^{deh}$ [%]	-	60 [%]
$T_{max}^{battery}$ [°C]	-	40 [°C]
$T_{set}^{deh}$ [°C]	-	18 [°C]
$W_{set}^{aircon}$	Automatic	$W^{air1}$(Mode 1)
		$W^{air2}$ (Mode 2)
*η* [%]	-	94 [%]

## Data Availability

Not applicable.

## Nomenclature

The following nomenclatures are used in this manuscript:

$Q_{in}$	The amount of heat generated by the ESS operation.
$Q_{out}$	The amount of heat transferred from the battery to the ESS container through the ESS operation.
P	The ESS output [W].
η	The ESS operating efficiency [%].
$R^{th}$	Thermal resistance [°C/W].
C	Heat capacity.
τ	Time constant.
$RH_{t}^{room}$	The relative humidity of the ESS container at time t [%].
$RH_{set}^{deh}$	The target relative humidity of the ESS container.
$AH_{t}^{room}$	The absolute humidity of the ESS container at time t [g/$m^{3}$].
$AH_{set}^{deh}$	The target absolute humidity of the ESS container [g/$m^{3}$].
$SMC_{T}$	The amount of saturated water vapor.
$T_{t}^{room}$	The indoor temperature of the ESS container at time t [℃].
$T_{set}^{room}$	The target indoor temperature of the ESS container [℃].
$T_{max}^{battery}$	The upper limit of the battery temperature [℃].
$T^{diff}$	Defining $\;T^{battery}-T^{room}$.
$T^{0}$	The initial temperature of the battery.
$T_{forcast-off}^{battery}$	The battery temperature at a steady state when the air conditioner is not operating [℃].
$T_{forcast-on}^{battery}$	The battery temperature at a steady state when the air conditioner is operating [℃].
$T_{set}^{deh}$	The lowest set temperature of the air conditioner [℃].
$T_{set}^{aircon}$	The set temperature of the air conditioner [℃].
$W_{set}^{aircon}$	The set wind speed of the air conditioner.
$W^{air1}$	The lowest wind speed of the air conditioner.
$W^{air2}$	The highest wind speed of the air conditioner.

## References

[1] Wang W., Yuan B., Sun Q., Wennersten R. (2022). Application of energy storage in integrated energy systems—A solution to fluctuation and uncertainty of renewable energy. J. Energy Storage.

[2] Hu Y., Armada M., Sánchez M.J. (2022). Potential utilization of battery energy storage systems (BESS) in the major European electricity markets. Appl. Energy.

[3] Pierro M., Perez R., Perez M., Moser D., Cornaro C. (2021). Imbalance mitigation strategy via flexible PV ancillary services: The Italian case study. Renew. Energy.

[4] Mahamud R., Park C. (2022). Theory and Practices of Li-Ion Battery Thermal Management for Electric and Hybrid Electric Vehicles. Energies.

[5] Ma S., Jiang M., Tao P., Song C., Wu J., Wang J., Deng T., Shang W. (2018). Temperature effect and thermal impact in lithium-ion batteries: A review. Prog. Nat. Sci. Mater. Int..

[6] Liu K., Wei Z., Zhang C., Shang Y., Teodorescu R., Han Q.L. (2022). Towards long lifetime battery: AI-based manufacturing and management. IEEE/CAA J. Autom. Sin..

[7] Xiong R., Li L., Tian J. (2018). Towards a smarter battery management system: A critical review on battery state of health monitoring methods. J. Power Sources.

[8] Liu K., Gao Y., Zhu C., Li K., Fei M., Peng C., Zhang X., Han Q.L. (2022). Electrochemical modeling and parameterization towards control-oriented management of lithium-ion batteries. Control Eng. Pract..

[9] Leng F., Tan C.M., Pecht M. (2015). Effect of Temperature on the Aging rate of Li Ion Battery Operating above Room Temperature. Sci. Rep..

[10] Olabi A.G., Maghrabie H.M., Adhari O.H.K., Sayed E.T., Yousef B.A., Salamah T., Kamil M., Abdelkareem M.A. (2022). Battery thermal management systems: Recent progress and challenges. Int. J. Thermofluids.

[11] Qin P., Sun J., Yang X., Wang Q. (2021). Battery thermal management system based on the forced-air convection: A review. ETransportation.

[12] IBM (2020). Installation and User Guide-Model 084.

[13] Electrical Energy Storage (EES) Systems; Part 4-3: The Protection Requirements of BESS According to the Environmental Conditions and Location Types. https://www.iec.ch/dyn/www/f?p=103:38:12520601337126::::FSP_ORG_ID,FSP_APEX_PAGE,FSP_PROJECT_ID:9463,23,103984.

[14] (2016). Energy Storage System Guide for Compliance with Safety Codes and Standards.

[15] Nezhad A.E., Rahimnejad A., Gadsden S.A. (2021). Home energy management system for smart buildings with inverter-based air conditioning system. Int. J. Electr. Power Energy Syst..

[16] You F., Qian Y., Liang J., Sun Y. (2017). Research on MW level containerized battery energy storage system. Chin. J. Power Sources.

[17] Xiong Z., Berquist J., Gunay H.B., Cruickshank C.A. (2021). An inquiry into the use of indoor CO_2_ and humidity ratio trend data with inverse modelling to estimate air infiltration. Build. Environ..

[18] Tanyer A.M., Tavukcuoglu A., Bekboliev M. (2018). Assessing the airtightness performance of container houses in relation to its effect on energy efficiency. Build. Environ..

[19] Back D.S., Lim K.B. (2021). Study on Analysis of Fire Factor and Development Direction of Standard/safety Requirement to Keep Safety for Energy Storage System (ESS). Turk. J. Comput. Math. Educ..

[20] Park K.M., Kim J.H., Park J.Y., Bang S.B. (2018). A Study on the Fire Risk of ESS through Fire Status and Field Investigation. Korean Inst. Fire Sci. Eng..

[21] Kim W., Kang S., Shin G. (2019). Study on Improvement of Dew Point within ESS Container for Fire Prevention. J. Korea Soc. Disaster Inf..

[22] Song J.W. (2021). Analysis of ESS (Electric Storage System) Electric Fire Causes, Countermeasures, and Safety Management Guidelines. J. Ind. Technol. Res..

[23] Jang H.j., Song T.S., Kim J.Y., Kim S.J., Jang T.H. (2019). A Study on The Safety Measures against Fire through an ESS Fire Analysis. Soc. Stand. Certif. Saf..

[24] Purwanto F.H., Utami E., Pramono E. Design of Server Room Temperature and Humidity Control System using Fuzzy Logic Based on Microcontroller. Proceedings of the 2018 International Conference on Information and Communications Technology (ICOIACT).

[25] Chun L., Gong G., Peng P., Wan Y., Chua K.J., Fang X., Li W. (2021). Research on thermodynamic performance of a novel building cooling system integrating dew point evaporative cooling, air-carrying energy radiant air conditioning and vacuum membrane-based dehumidification (DAV-cooling system). Energy Convers. Manag..

[26] Jingyun L., Ping L. Temperature and humidity control with a model predictive control method in the air-conditioning system. Proceedings of the 2017 International Conference on Advanced Mechatronic Systems (ICAMechS).

[27] Daskalov P.I., Arvanitis K.G., Pasgianos G.D., Sigrimis N.A. (2006). Non-linear Adaptive Temperature and Humidity Control in Animal Buildings. Biosyst. Eng..

[28] Zhang T., Liu X., Jiang Y. (2014). Development of temperature and humidity independent control (THIC) air-conditioning systems in China—A review. Renew. Sustain. Energy Rev..

[29] Xu X., Zhong Z., Deng S., Zhang X. (2018). A review on temperature and humidity control methods focusing on air-conditioning equipment and control algorithms applied in small-to-medium-sized buildings. Energy Build..

[30] Chen X., Wang X., Sun W., Jiang C., Xie J., Wu Y., Jin Q. (2023). Integrated interdigital electrode and thermal resistance micro-sensors for electric vehicle battery coolant conductivity high-precision measurement. J. Energy Storage.

[31] Wei Z., Hu J., He H., Yu Y., Marco J. (2022). Embedded Distributed Temperature Sensing Enabled Multistate Joint Observation of Smart Lithium-Ion Battery. IEEE Trans. Ind. Electron..

[32] Semeraro C., Olabi A.G., Aljaghoub H., Alami A.H., Al Radi M., Dassisti M., Abdelkareem M.A. (2023). Digital twin application in energy storage: Trends and challenges. J. Energy Storage.

[33] Qin Y., Arunan A., Yuen C. (2023). Digital Twin for Real-time Li-Ion Battery State of Health Estimation with Partially Discharged Cycling Data. IEEE Trans. Ind. Inform..

[34] Tran M.K., Panchal S., Khang T.D., Panchal K., Fraser R., Fowler M. (2022). Concept Review of a Cloud-Based Smart Battery Management System for Lithium-Ion Batteries: Feasibility, Logistics, and Functionality. Batteries.

[35] Li W., Rentemeister M., Badeda J., Jöst D., Schulte D., Sauer D.U. (2020). Digital twin for battery systems: Cloud battery management system with online state-of-charge and state-of-health estimation. J. Energy Storage.

[36] Yang S., Zhang Z., Cao R., Wang M., Cheng H., Zhang L., Jiang Y., Li Y., Chen B., Ling H. (2021). Implementation for a cloud battery management system based on the CHAIN framework. Energy AI.

[37] Promotion of ‘Energy Storage System (ESS) Safety Enhancement Measures’ to Protect People’s Lives and Property. https://www.motie.go.kr/motie/ne/presse/press2/bbs/bbsView.do?bbs_seq_n=165570&bbs_cd_n=81&currentPage=1&search_key_n=&cate_n=1&dept_v=&search_val_v=.

[38] Han X., Zhang X. (2011). Experimental study on a residential temperature–humidity separate control air-conditioner. Energy Build..

[39] Hwang J., Seo H., Byeon N., Joo Y., Kim J. (2015). An Experimental Study on Dehumidifying Efficiency and Thermal Comfort Improvement for the Inverter-driven Residential Air Conditioner. Proc. SAREK.

[40] Szekely V., Rencz M. (2000). Thermal dynamics and the time constant domain. IEEE Trans. Compon. Packag. Technol..

